# Perceived Social Support Among Patients with Permanent Cardiac Pacemakers

**DOI:** 10.19102/icrm.2026.17106

**Published:** 2026-10-15

**Authors:** Eirini Stavrou, Dionyssios Leftheriotis, Panagiota Flevari, Maria Polikandrioti

**Affiliations:** 1 Department of Nursing, University of West Attica, Egaleo, Greece

**Keywords:** Pacemaker, quality of life, social support

## Abstract

In recent years, the number of implanted permanent cardiac pacemakers (PPMs) has been steadily increasing. Perceived social support is a source that enhances device acceptance, overall well-being, and quality of life among PPM recipients. This study aimed to explore perceived social support and its associated factors. In this cross-sectional study, 120 patients with PPMs were enrolled. Data were collected through interviews using the Multidimensional Scale of Perceived Social Support (MSPSS) for assessing perceived social support from family, significant others, and friends. Patient characteristics were also recorded. Statistical significance was defined as *P* < .05. The majority of participants were men (58.3%) and had their pacemaker implanted for >5 years (46.7%). The median scores on the MSPSS subscales were 28 points for significant others, 28 points for family, and 16 points for friends, indicating high levels of perceived social support within the possible score range of 4–28 points. Scores on the MSPSS were high for significant others (mean ± standard deviation, 27.2 ± 2.7 points; range, 4–28 points) and family (27.1 ± 2.7 points; range, 4–28 points), while support from friends was comparatively lower (16.7 ± 8.6 points; range, 4–28 points). Statistically significant associations were observed between support from significant others and both age (*P* = .049) and marital status (*P* = .010). Family support was significantly associated with marital status (*P* = .007), years since pacemaker implantation (*P* = .027), and patients’ self-reported level of health information (*P* = .017). Support from friends was significantly associated with age (*P* = .045), marital status (*P* = .012), comorbidity (*P* = .028), and device acceptance (*P* = .025). In conclusion, higher perceived social support was reported from family and significant others compared with friends. Marital status, age, comorbidity, years since pacemaker implantation, patients’ level of health information, comorbidity, and device acceptance were associated with social support levels.

**CLINICAL IMPACT:**

This study provides important insights into the social support experienced by patients with permanent pacemakers. While support from significant others and family was high, support from friends was lower, highlighting differences in the sources of social support. Importantly, social support was associated with key patient characteristics, including age, marital status, comorbidity, years since implantation, perceived health information, and device acceptance. These findings suggest that social support is a multidimensional aspect of pacemaker care and that some patients may be more vulnerable to reduced support. Recognizing recipients support needs and strengthening communication, patient and family education, and social connectedness may facilitate better adaptation to the pacemaker and promote patients’ overall well-being. The findings highlight the need to incorporate assessment of social support into patient-centered pacemaker follow-up and nursing care.

## Introduction

Permanent cardiac pacemakers (PPMs) are small, implantable electronic devices used to treat heart rhythm disorders. According to global estimates, about 3 million individuals live with a PPM, while approximately 600,000 new devices are implanted each year.^1^ The European Heart Rhythm Association (EHRA) in 2017 reported a 20% increase in PPM implantations over a 10-year period.^2^ Subsequently, in 2025, the EHRA reported that PPM implantations (first time and replacements) had reached 739 per million people across European Society of Cardiology (ESC) member countries.^3^

Since the first pacemaker implantation by Elmquist and Senning at Karolinska Hospital in 1958, cardiac pacing has advanced considerably, with improvements in implantation techniques, device software, battery longevity, and remote monitoring.^4^ PPM implantation corrects bradycardia, normalizes heart rate, and enhances hemodynamics, supporting better daily functioning.^5^ Nevertheless, recipients must adjust to lifestyle changes, adhere to medications, attend follow-up visits, and manage potential complications, highlighting the need for ongoing support.^6,7^

Social support refers to an individual’s informal and formal sources of support (family, friends, and significant others) and is categorized into received and perceived support. Perceived support is defined as an individual’s perception of help being available when needed, while received support refers to the actual help provided to an individual. Notably, perceived social support has been shown to be a stronger predictor of well-being than received support.^8,9^ According to the Health Promotion Model, the external system of perceived social support is predictive of health empowerment and better overall health, as individuals who perceive greater support are more likely to exert control over disease, promote health through acquisition of knowledge and skills, enhance self-management, and modify health behaviors.^10^ Conversely, among individuals with cardiac disorders, suboptimal perceived social support is associated with adverse outcomes such as psychological distress, loneliness, depression, increased risk of hospital admission, and frequent visits to emergency departments.^11^

In the field of pacing, particularly in the post-implantation phase, social support improves device acceptance among elderly PPM recipients^9^; peer support improves self-care, follow-up attendance, and psychological state compared to conventional care^12^; and a patient network of the same group provides necessary information support to overcome several challenges, including device adaptation, navigation to health care, and psychological burden.^13^ The 2021 ESC guidelines on cardiac pacing and cardiac resynchronization therapy recognize the patient’s needs, beliefs, expectations, preferences, goals, and values and place emphasis on patient-centered care and family support.^14^ Patients frequently experience psychological stress following pacemaker implantation, highlighting the challenges associated with adjusting to having a permanent cardiac device. Psychological adaptation represents a multifaceted and evolving process that encompasses emotional adjustment in conjunction with the implementation of adaptive coping strategies, both of which contribute to the successful integration of the device into patients’ daily functioning.^15^

Despite the recognized benefits of PPM implantation, research on perceived social support among recipients remains limited. This represents a critical gap, as understanding psychosocial factors is essential for optimizing patient-centered care. The present study addresses this gap by examining perceived social support among PPM recipients.

The objective of the current study was to determine (1) levels of perceived social support and (2) factors associated with social support.

## Materials and methods

### Design, setting, and period of the study

In this cross-sectional, single-center study, 120 patients with implanted PPMs who attended the outpatient clinic of a public hospital for conventional in-person follow-up between February 2024 and June 2025 were enrolled. A total of 150 patients were approached, of whom 120 consented to participate.

Participants were selected using convenience sampling, meaning enrollment was based on their proximity and availability rather than a random selection process (ie, a non-probability sampling method). This approach is well established in health care research and cardiovascular nursing, although it carries inherent risks of bias and limits the generalizability of the findings.^16,17^ No a priori power calculation was performed, as the study was exploratory in nature and included all eligible patients available during the study period. The sample size was constrained by patient availability, and the study aimed primarily to describe associations rather than to test a predefined hypothesis. To support interpretation, the magnitude of associations was reported using regression coefficients (β) with corresponding 95% confidence intervals (CIs), providing effect-size estimates that highlight the clinical relevance of the findings. 

### Inclusion and exclusion criteria of the sample

The criteria for inclusion in the study were: (1) understanding of the Greek language; (2) ability to comprehend, read, and sign the informed consent form; and (3) regular attendance of follow-up, as scheduled by health professionals. The exclusion criteria were: (1) insufficient communication abilities, (2) severe symptoms of distress during follow-up, and (3) sensory impairment (hearing or sight). The exclusion criteria identified from the medical records or by the cardiologist were: (1) diagnosis of a psychiatric illness, (2) receiving medical treatment for a psychiatric disorder, and (3) diagnosis of cognitive impairment.

Of note, the exclusion of patients with psychiatric disorders or cognitive impairments may have systematically removed individuals who are more likely to experience lower levels of perceived social support. Consequently, this selection criterion could introduce bias and limit the generalizability of the findings to the broader population of patients with pacemakers. On the other side of the spectrum, patients with psychiatric disorders or cognitive impairments have difficulty in understanding or accurately responding to questionnaire items, which could introduce measurement error. Additionally, psychiatric conditions may independently affect perceptions of social support, potentially confounding the associations under investigation. Excluding these patients also addresses ethical considerations, as individuals with significant cognitive impairment may be unable to provide fully informed consent.

### Data collection and procedure

Data collection was conducted for each participant after the monitoring and follow-up period, lasting approximately 15–20 min per patient. Data were collected through interviews in a private office at the outpatient clinics to avoid missing data and ensure confidentiality.

### Research instrument

Data collection was carried out using the standardized scale, the Multidimensional Scale of Perceived Social Support (MSPSS), to assess the social support perceived by patients. Data collected for each patient included demographic and clinical characteristics as well as other self-reported variables, which were selected through clinical observations and literature review.

### Assessment of perceived social support

To assess the perceived social support of participants, the MSPSS was used. The MSPSS consists of 12 items evaluating the level of social support perceived by patients from friends, family, and significant others. Respondents answered each item using a 7-point Likert scale. Each response option is scored from 1 to 7 points; the scores for each group of items are then summed, yielding separate support scores from friends, family, and significant others, with higher scores indicating greater levels of perceived social support.^18^ This scale was tested in a Greek population by Theofilou,^19^ yielding an overall Cronbach’s α of 0.80, ranging between 0.74 (for the friends subscale) and 0.78 (for both the family and significant others subscales). The test–retest reliability intraclass correlation coefficient was 0.894. It is worth noting that people considered as significant others are dating partners, coworkers, neighbors, spiritual advisors, health care personnel, or peer groups.^19^

### Ethical considerations

The present study was approved by the research committee of the public hospital (approval no.: 117, approved February 4, 2025). Participants were informed by the researcher about the purposes of the study and their written informed consent was obtained. Anonymity and confidentiality was guaranteed during data collection. All subjects were informed of their right to refuse or discontinue participation in the study, according to the ethical standards of the Declaration of Helsinki (1989) of the World Medical Association.

### Statistical analysis

Categorical data are presented as absolute and relative frequencies (%), while continuous data are presented as mean, standard deviation, median, and interquartile range values. The *t* test and analysis of variance, or the Kruskal–Wallis and Mann–Whitney *U* tests, were used to examine the association between perceived social support and patients’ characteristics, depending on whether the data followed a normal distribution or not. Normality was assessed using the Shapiro–Wilk test and visually through histograms. Multiple linear regression analysis was used to estimate the effect of patients’ characteristics on perceived social support. Results are presented as β regression coefficients and 95% CIs. A *P* value of <.05 was considered statistically significant. All statistical analyses were performed using SPSS software, version 25 (IBM Corp., Armonk, NY, USA).

## Results

### Sample description

According to **Table 1**, the majority of participants were men (58.3%), were aged >75 years (80.8%), were married (63.4%), had primary education (53.3%), were retired (90.8%), resided in Athens (75.0%), and had two children (59.2%).

**Table 1:** Description of Demographic Characteristics (N = 120)

	n (%)
Sex	
Male	70 (58.3%)
Female	50 (41.7%)
Age	
<75 years	23 (19.2%)
75–84 years	61 (50.8%)
≥85 years	36 (30.0%)
Marital status	
Married	76 (63.4%)
Single	3 (2.5%)
Divorced	4 (3.3%)
Widowed	37 (30.8%)
Educational level	
Primary education	64 (53.3%)
Secondary education	34 (28.3%)
University	22 (18.4%)
Occupation	
Retired	109 (90.8%)
Employed	8 (6.7%)
Household	3 (2.5%)
Place of residence	
Athens	90 (75.0%)
Regional capital	3 (2.5%)
Province	27 (22.5%)
Number of children	
0	6 (5.0%)
1	14 (11.7%)
2	71 (59.2%)
>2	29 (24.1%)

According to **Table 2**, 84.2% of participants had a DDD pacing mode and 46.7% had their pacemaker for >5 years. Regarding the causes for pacemaker implantation, 40.8% had atrioventricular block, 40.0% had sick sinus syndrome, and 12.5% had bradycardic atrial fibrillation (AF), while 75.0% had a comorbidity and 70.9% were classified as New York Heart Association class I. In addition, 50.0% had experienced syncope prior to pacemaker implantation, while 1.7% reported syncope after implantation. Pacemaker replacement (once or twice) had been performed in 22.5% of participants. The majority of patients stated that they accepted the PPM device (42.5%) and reported being “very or fairly” well informed about their health (88.3%).

**Table 2:** Description of Clinical and Other Self-reported Characteristics (N = 120)

	n (%)
Pacing mode^a^	
VVI	19 (15.8%)
DDD	101 (84.2%)
Years since implantation	
1 year	29 (24.1%)
2–4 years	35 (29.2%)
≥5 years	56 (46.7%)
Indication for implantation	
Heart failure	8 (6.7%)
Atrioventricular block	49 (40.8%)
Sick sinus syndrome	48 (40.0%)
Bradycardic atrial fibrillation	15 (12.5%)
Other comorbidities (yes)	90 (75.0%)
NYHA class	
I	85 (70.9%)
II	35 (29.1%)
Syncope before implantation (yes)	60 (50.0%)
Syncope after implantation (yes)	2 (1.7%)
Pacemaker replacement(s)	
None	93 (77.5%)
One	25 (20.8%)
Two	2 (1.7%)
Acceptance of PPM	
Very much	51 (42.5%)
Fairly	36 (30.0%)
A little	25 (20.8%)
Not at all	8 (6.7%)
Level of information on health	
Very well informed	52 (43.3%)
Fairly well informed	54 (45.0%)
Poorly informed	14 (11.7%)

*Abbreviations:* NYHA, New York Heart Association; PPM, permanent cardiac pacemaker. ^a^PPM with one lead: ventricular-inhibited VVI or two leads: dual-chamber DDD.

### Perceived social support of permanent cardiac pacemaker recipients

According to the results presented in **Table 3**, which refers to the social support perceived by patients, it was observed that at least 50% of patients scored above 28, 28, and 16 points (medians) for support from significant others, family, and friends, respectively. Additionally, 25% of the patients scored above 28, 28, and 24 points, respectively. The mean scores were 27.2 ± 2.7 points for support from significant others, 27.1 ± 2.7 points for support from family, and 16.7 ± 8.6 points for support from friends. These values, considering the possible score range of 4–28 points, indicate high levels of social support as perceived by patients **(Figure 1)**. Furthermore, the results suggest that patients experience greater support from significant others and family, and less support from friends.

**Table 3:** Levels of Perceived Social Support (N = 120)

Support from	Mean (SD), points	Median (IQR), points
Significant others (range: 4–28 points)	27.2 (2.7)	28.0 (28.0–28.0)
Family (range: 4–28 points)	27.1 (2.7)	28.0 (28.0–28.0)
Friends (range: 4–28 points)	16.7 (8.6)	16.0 (8.0–24.0)

*Abbreviations:* IQR, interquartile range; SD, standard deviation.

**Figure 1:**
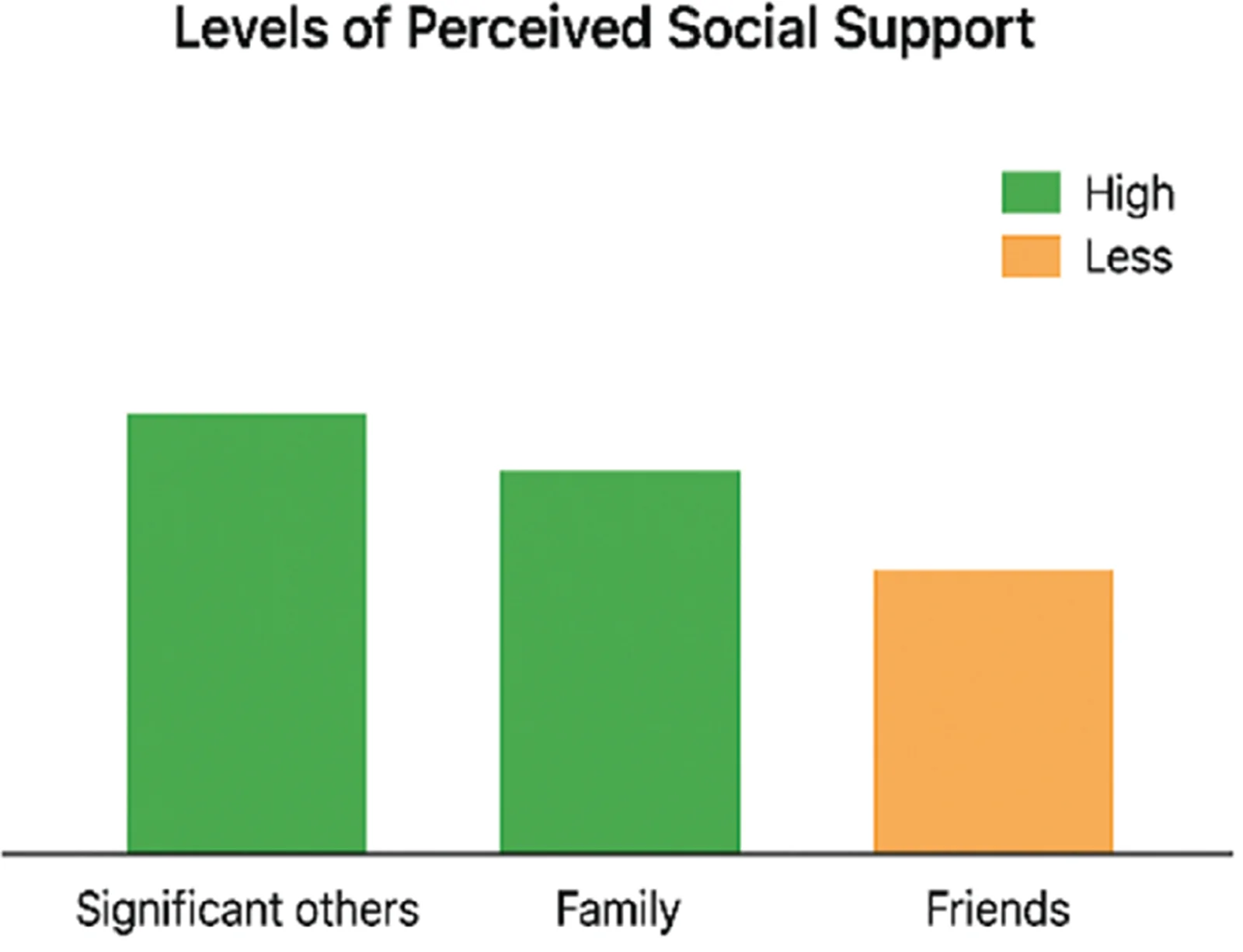
Perceived social support.

The median score for both the significant others and family subscales was 28 points, the maximum possible score, indicating high levels of perceived social support. This clustering at the upper limit suggests a potential ceiling effect, which may reduce variability and limit detection of differences.

### Factors associated with perceived social support

**Tables 4 and 5** present the associations between perceived social support and patients’ characteristics.

**Table 4:** Association of Support from Significant Others and Family with Patients’ Characteristics

	Support from Significant Others			Support from Family		
	Mean (SD)	Median (IQR)	*P* Value	Mean (SD)	Median (IQR)	*P* Value
Sex			.081			.163
Male	27.5 (2.2)	28.0 (28.0–28.0)		27.5 (1.7)	28.0 (28.0–28.0)	
Female	26.8 (3.2)	28.0 (28.0–28.0)		26.7 (3.4)	28.0 (28.0–28.0)	
Age			.049			.466
<75 years	27.3 (3.1)	28.0 (28.0–28.0)		27.5 (1.3)	28.0 (28.0–28.0)	
75–84 years	27.4 (2.3)	28.0 (28.0–28.0)		27.2 (2.7)	28.0 (28.0–28.0)	
≥85 years	26.6 (3.1)^a^	28.0 (27.5–28.0)		26.6 (3.2)	28.0 (28.0–28.0)	
Marital status			.010			.007
Married	27.8 (0.9)	28.0 (28.0–28.0)		27.7 (1.2)	28.0 (28.0–28.0)	
Single/divorced	24.7 (6.0)^a^	28.0 (20.0–28.0)		25.4 (3.9)^a^	27.0 (22.0–28.0)	
Widowed	26.4 (3.7)	28.0 (28.0–28.0)		26.3 (4.0)	28.0 (28.0–28.0)	
Educational level			.218			.350
Primary education	27.2 (2.6)	28.0 (28.0–28.0)		27.0 (3.0)	28.0 (28.0–28.0)	
Secondary education	26.7 (3.5)	28.0 (28.0–28.0)		27.0 (2.8)	28.0 (27.0–28.0)	
University	27.9 (0.6)	28.0 (28.0–28.0)		27.7 (0.8)	28.0 (28.0–28.0)	
Occupation			.262			.560
Retired	27.2 (2.5)	28.0 (28.0–28.0)		27.2 (2.4)	28.0 (28.0–28.0)	
Employed	28.0 (0.0)	28.0 (28.0–28.0)		27.9 (0.4)	28.0 (28.0–28.0)	
Household	23.0 (8.7)	28.0 (13.0–28.0)		22.7 (9.2)	28.0 (12.0–28.0)	
Place of residence			.300			.338
Athens/regional capital	27.4 (2.1)	28.0 (28.0–28.0)		27.4 (2.2)	28.0 (28.0–28.0)	
Province	26.3 (4.1)	28.0 (28.0–28.0)		26.2 (3.8)	28.0 (28.0–28.0)	
Number of children			.856			.517
0	26.0 (4.9)	28.0 (28.0–28.0)		24.3 (5.7)	28.0 (18.0–28.0)	
1	26.9 (4.0)	28.0 (28.0–28.0)		27.9 (0.4)	28.0 (28.0–28.0)	
2	27.4 (1.9)	28.0 (28.0–28.0)		27.3 (1.9)	28.0 (28.0–28.0)	
>2	27.1 (3.2)	28.0 (28.0–28.0)		26.8 (3.6)	28.0 (28.0–28.0)	
Pacing mode			.365			.394
VVI	27.4 (2.1)	28.0 (28.0–28.0)		27.4 (2.5)	28.0 (28.0–28.0)	
DDD	27.1 (2.8)	28.0 (28.0–28.0)		27.1 (2.7)	28.0 (28.0–28.0)	
Years since implantation			.168			.027
≤1 year	27.5 (2.8)	28.0 (28.0–28.0)		28.0 (0.2)	28.0 (28.0–28.0)	
2–4 years	26.8 (2.9)	28.0 (28.0–28.0)		26.5 (3.1)^a^	28.0 (27.0–28.0)	
≥5 years	27.3 (2.6)	28.0 (28.0–28.0)		27.0 (3.0)	28.0 (28.0–28.0)	
Indication for implantation			.603			.427
Heart failure	28.0 (0.0)	28.0 (28.0–28.0)		28.0 (0.0)	28.0 (28.0–28.0)	
Atrioventricular block	26.6 (3.8)	28.0 (28.0–28.0)		26.7 (3.5)	28.0 (28.0–28.0)	
Sick sinus syndrome	27.6 (1.3)	28.0 (28.0–28.0)		27.4 (1.7)	28.0 (28.0–28.0)	
Bradycardic AF	27.3 (2.3)	28.0 (28.0–28.0)		27.2 (2.8)	28.0 (28.0–28.0)	
Comorbidities			.424			.675
Yes	27.1 (2.8)	28.0 (28.0–28.0)		27.1 (2.7)	28.0 (28.0–28.0)	
No	27.5 (1.8)	28.0 (28.0–28.0)		27.3 (2.1)	28.0 (28.0–28.0)	
NYHA			.746			.765
I	27.2 (2.8)	28.0 (28.0–28.0)		27.1 (2.7)	28.0 (28.0–28.0)	
II/III	27.0 (2.7)	28.0 (28.0–28.0)		27.0 (2.8)	28.0 (28.0–28.0)	
Syncope before PPM			.271			.640
Yes	27.0 (3.0)	28.0 (28.0–28.0)		26.8 (3.2)	28.0 (28.0–28.0)	
No	27.4 (2.4)	28.0 (28.0–28.0)		27.5 (1.9)	28.0 (28.0–28.0)	
Pacemaker replacements			.444			.966
None	27.1 (2.9)	28.0 (28.0–28.0)		27.0 (2.9)	28.0 (28.0–28.0)	
1–2	27.4 (1.8)	28.0 (28.0–28.0)		27.4 (1.5)	28.0 (28.0–28.0)	
Acceptance of PPM			.587			.133
Very much	27.7 (1.4)	28.0 (28.0–28.0)		27.6 (1.7)	28.0 (28.0–28.0)	
Fairly	27.0 (3.5)	28.0 (28.0–28.0)		27.3 (2.8)	28.0 (28.0–28.0)	
A little/not at all	26.7 (3.2)	28.0 (28.0–28.0)		26.2 (3.5)	28.0 (27.0–28.0)	
Level of information on health			.426			.017
Very well informed	27.5 (2.4)	28.0 (28.0–28.0)		27.7 (1.6)	28.0 (28.0–28.0)	
Fairly well informed	27.1 (2.7)	28.0 (28.0–28.0)		27.0 (2.9)	28.0 (28.0–28.0)	
Poorly informed	26.3 (3.8)	28.0 (28.0–28.0)		25.4 (4.2)^a^	28.0 (24.0–28.0)	

*Abbreviations:* AF, atrial fibrillation; IQR, interquartile range; NYHA, New York Heart Association; PPM, permanent cardiac pacemaker; SD, standard deviation. ^a^Statistically significantly different score from the other categories after Bonferroni correction for multiple comparisons.

**Table 5:** Association of Support from Friends with Patients’ Characteristics

	Support from Friends		
	Mean (SD)	Median (IQR)	*P* Value
Sex			.123
Male	17.8 (8.5)	20.0 (10.0–24.0)	
Female	15.5 (8.7)	16.0 (4.0–24.0)	
Age			.045
<75 years	20.1 (8.8)	24.0 (16.0–28.0)	
75–84 years	16.4 (8.6)^a^	16.0 (8.0–24.0)	
≥85 years	14.9 (8.2)^a^	16.0 (8.0–21.5)	
Marital status			.012
Married	18.0 (8.1)	20.0 (11.0–24.0)	
Single/divorced	19.6 (11.0)	24.0 (4.0–28.0)	
Widowed	13.3 (8.5)^a^	12.0 (4.0–20.0)	
Educational level			.258
Primary education	15.5 (8.9)	16.0 (8.0–24.0)	
Secondary education	17.5 (8.3)	20.0 (12.0–24.0)	
University	19.0 (8.0)	22.0 (16.0–24.0)	
Occupation			.055
Retired	16.1 (8.6)	16.0 (8.0–24.0)	
Employed	23.0 (4.9)	24.0 (18.5–27.5)	
Household	20.7 (11.0)	26.0 (8.0–28.0)	
Place of residence			.867
Athens/regional capital	16.8 (8.4)	16.0 (8.0–24.0)	
Province	16.3 (9.5)	16.0 (4.0–24.0)	
Number of children			.412
0	16.0 (10.7)	16.0 (4.0–28.0)	
1	13.4 (8.6)	15.5 (4.0–20.0)	
2	17.3 (8.7)	20.0 (8.0–24.0)	
>2	16.9 (8.0)	16.0 (10.0–24.0)	
Pacing mode			.120
VVI	19.8 (6.4)	22.0 (16.0–24.0)	
DDD	16.1 (8.9)	16.0 (8.0–24.0)	
Years since implantation			.089
≤1 year	13.6 (9.1)	16.0 (4.0–20.0)	
2–4 years	17.5 (8.5)	21.0 (8.0–24.0)	
≥5 years	17.8 (8.2)	20.0 (11.0–24.0)	
Indication for implantation			.320
Heart failure	18.0 (11.7)	24.0 (4.0–28.0)	
Atrioventricular block	16.7 (8.2)	16.0 (8.0–24.0)	
Sick sinus syndrome	15.4 (8.9)	16.0 (6.0–24.0)	
Bradycardic atrial fibrillation	20.0 (7.0)	22.0 (16.0–25.0)	
Other comorbidities			.028
Yes	16.0 (8.7)	16.0 (8.0–24.0)	
No	21.5 (6.5)	22.0 (16.0–27.0)	
NYHA			.469
I	17.1 (8.4)	20.0 (9.0–24.0)	
II/III	15.4 (9.5)	16.0 (4.0–24.0)	
Syncope before implantation			.785
Yes	16.4 (8.8)	16.0 (8.0–24.0)	
No	17.0 (8.5)	18.0 (9.0–24.0)	
Pacemaker replacements			.208
None	16.2 (8.6)	16.0 (8.0–24.0)	
1–2	18.3 (8.8)	20.0 (10.0–26.0)	
Acceptance of PPM			.025
Very much	18.3 (8.7)	20.0 (10.0–26.0)	
Fairly	17.3 (8.2)	20.0 (8.5–24.0)	
A little/not at all	13.4 (8.3)^a^	15.0 (4.0–20.0)	
Level of information on health			.200
Very well informed	18.0 (8.9)	21.5 (8.0–25.0)	
Fairly well informed	16.1 (8.1)	16.0 (8.0–24.0)	
Poorly informed	14.1 (9.2)	16.0 (4.0–24.0)	

*Abbreviations:* IQR, interquartile range; NYHA, New York Heart Association; PPM, permanent cardiac pacemaker; SD, standard deviation. ^a^Statistically significantly different score from the other categories after Bonferroni correction for multiple comparisons.

A statistically significant association was observed between support from significant others **(Table 4)** and age (*P* = .049) as well as marital status (*P* = .010).

A significant association was also found between family support **(Table 5)** and marital status (*P* = .007), years since pacemaker implantation (*P* = .027), and patients’ self-reported level of information about their health (*P* = .017). Regarding support from friends **(Table 5)**, significant associations were found with age (*P* = .045), marital status (*P* = .012), presence of comorbidity (*P* = .028), and acceptance of pacemaker implantation (*P* = .025).

### Effect of patients’ characteristics on perceived social support

A multiple linear regression analysis was conducted to estimate the effect of patients’ characteristics (independent variables) on perceived social support (dependent variable) **(Table 6)**. Factors that were statistically significantly associated with perceived social support at the univariate level (*P* < .05) were entered in the models. The assumptions of normality and homoscedasticity were held.

**Table 6:** Effect of Patients’ Characteristics on Perceived Social Support

	Significant Others		Family		Friends	
	β Coeff (95% CI)	*P* Value	β Coeff (95% CI)	*P* Value	β Coeff (95% CI)	*P* Value
Age						
<75 years	Ref.		—		Ref.	
75–84 years	−0.05 (−1.36 to 1.25)	.934	—		−0.98 (−5.35 to 3.39)	.656
≥85 years	−0.70 (−2.14 to 0.74)	.340	—		−2.14 (−6.86 to 2.59)	.372
Marital status						
Married	Ref.		Ref.		Ref.	
Single/divorced	−3.19 (−5.26 to −1.12)	.003	−2.39 (−4.35 to −0.44)	.017	0.80 (−5.77 to 7.37)	.810
Widowed	−1.34 (−2.40 to −0.28)	.014	−1.31 (−2.30 to −0.32)	.010	−3.72 (−7.11 to −0.33)	.032
Years since implantation						
≤1 year	—		Ref.		—	
2–4 years	—		−1.39 (−2.63 to −0.15)	.029	—	
≥5 years	—		−0.69 (−1.82 to 0.45)	.232	—	
Other comorbidities (no vs. yes)	—				3.73 (−1.10 to 8.55)	.129
Acceptance of PPM						
Very much	—		—		Ref.	
Fairly	—		—		−0.83 (−4.44 to 2.78)	.651
A little/not at all	—		—		−4.93 (−8.62 to −1.25)	.009
Level of information on health						
Very well informed	—		Ref.		—	
Fairly well informed	—		−0.53 (−1.50 to 0.44)	.278	—	
Poorly informed	—		−1.99 (−3.48 to −0.50)	.009	—	

*Abbreviations:* β coeff, regression coefficient; CI, confidence interval; PPM, permanent cardiac pacemaker; Ref., reference category.

Regarding support from significant others, it was observed that single/divorced patients and widowed patients had significantly lower support scores compared to married patients, by 3.2 and 1.3 points, respectively (β = −3.19; 95% CI, −5.26 to −1.12; *P* = .003 and β = −1.34; 95% CI, −2.40 to −0.28; *P* = .014).

As for support from family, single/divorced patients and widowed patients had 2.4- and 1.3-point lower scores compared to married patients, respectively (β = −2.39; 95% CI, −4.35 to −0.44; *P* = .017 and β = −1.31; 95% CI, −2.30 to −0.30; *P* = .010). In addition, patients who had their pacemaker implanted for 2–4 years had significantly lower family support scores (by 1.4 points) compared to those who had it implanted for <1 year (β = −1.39; 95% CI, −2.63 to −0.15; *P* = .029). Furthermore, patients who felt poorly informed about their health condition reported 2-point lower family support scores compared to those who felt very well informed (β = −1.99; 95% CI, −3.48 to −0.50; *P* = .009).

Concerning support from friends, widowed patients had 3.7-point lower scores than married patients (β = −3.72; 95% CI, −7.11 to −0.33; *P* = .032). Moreover, patients who felt that their life depended little on their pacemaker had 5-point lower friend support scores compared to those who felt their life depended heavily on it (β = −4.93; 95% CI, −8.63 to −1.25; *P* = .009).

## Discussion

The current study aimed to explore levels of perceived social support from family, friends, and significant others, as well as the factors associated with it. To the best of our knowledge, evidence on social support as perceived by PPM recipients is limited. Therefore, the discussion interprets perceived social support in the context of chronic illness. Perceived social support plays a crucial role in helping patients with chronic illnesses adapt to their conditions, enhance coping strategies, and maintain motivation for disease management.^6^ It facilitates self-care by supporting decision-making and help-seeking behaviors,^20^ provides a sense of security and confidence in overcoming illness, helps patients feel valued,^8,9^ protects against mental health disorders,^21,22^ and enhances subjective well-being.^23^ Additionally, the benefits of social support extend to caregivers, with family support associated with better mental quality of life (QoL) and friend support linked to better physical QoL within the patient–caregiver dyad.^8^

Regarding age, patients aged >85 years reported lower support from significant others and patients aged <75 years reported higher support from friends. Although fewer social relationships are observed among individuals of advanced age, they are more intimate and have a stable presence in their lives.^9^ Interestingly, as age advances, individuals tend to prioritize close relationships and are more unwilling to reveal themselves to significant others or even more others may underestimate their needs, attributing them to aging.^24,25^ According to the socioemotional selectivity theory, older adults pay more attention to close, emotional connections and those providing meaningful support to their well-being.^25^ Possibly, participants aged <75 years reported more support from friends, as they are still independent in performing daily activities or going outdoors and have better well-being and an optimal perception of their health state.^25^ Patients in better physical condition are able to spend more time with friends and therefore feel more supported by them.^8^ Furthermore, greater social support may be associated with better cognitive abilities.^26^ A notable finding of the present study is that single or divorced patients experienced less support from significant others and family, whereas they reported more support from friends. Previous research suggests that single individuals may rely more on friendships for practical and emotional support^27^; however, the reasons for these patterns were not directly assessed in the present study and require further investigation.^27^

Patients with comorbid conditions reported lower support from friends. This group of patients may encounter difficulties in maintaining relationships outside the family context due to functional impairment and psychological distress related to comorbidity.^8^

Discomfort and restrictions after implantation might be an additional barrier to maintaining social interactions.^28^ Individuals with multiple chronic illnesses present with greater disability, higher mortality rates, increased health care costs, and poorer QoL. Considering the increased coexistence of chronic illnesses due to the aging of the population and the global rise in life expectancy,^8^ enhancing support from friends remains particularly important in the context of comorbidity.

Participants who had accepted the implanted cardiac device reported more support from friends. Based on the current findings, there is a need to gain better insight into the experiences of PPM recipients. Patients with intense symptom burden or poor pre-implantation health may perceive the implanted device as a “valuable tool” to improve health, thus demonstrating a more positive perception of the device.^19,29^

De Bardi et al.^9^ found that, 30 days after PPM implantation, increases in social support, device acceptance, and QoL were reported among 62 participants (median age, 76.5 years). The study further identified higher QoL, greater perceived social support, and implantation for sinus node disease or AF with slow ventricular rate as independent predictors of device acceptance. Higher perceived social support was associated with greater cardiac device acceptance, even after adjusting for QoL, the primary mediator of social support.^9^

Patients who felt poorly informed about their health reported less support from family. This may reflect limited health literacy, family conflicts, or reduced access to health care services. Inadequate information provision by health professionals can exacerbate perceived social isolation, which in turn may increase the risk of depressive symptoms, cognitive decline, sleep disturbances, and overall morbidity.^30^

Misunderstandings or misconceptions arising from outdated or insufficient information contribute to unrealistic expectations.^31^ Intriguingly, PPM recipients prefer to receive elaborate information about pacemakers, including information about device withdrawal, early in the pre-implantation phase. Therefore, health care professionals should ensure that patients understand recommendations while also addressing patients’ concerns.^31^ Nurses facilitate patients to express their needs by encouraging open discussion. According to ESC Guidelines on cardiac pacing, nurses are recommended to enhance PPM-related knowledge among both patients and their families.^15^ Development of effective interventions and clinical guidelines that address social support as perceived by PPM recipients requires not only more knowledge about their perceptions but also evaluation of the support source, as it may be associated with negative interactions such as criticism or conflicts.^31^

It is of crucial importance to consider confounding factors when interpreting the current results. In the present study, 80.8% of participants were aged >75 years, which may introduce bias. Age influences social resources and, consequently, social support.

Advanced age is associated with a more positive life perception and better adaptation to losses as well as reduced expectations regarding life expectancy.^24^ Of note, the younger recipient group (41–50 years) raises more concerns about social support, as they may bear greater economic burdens.^32^ A better understanding of perceived social support requires exploring differences across age groups.

Furthermore, in order to avoid disparities, the study included only recipients of transvenous pacemakers and not leadless ones. Leadless pacemakers were developed to reduce complications associated with transvenous devices, particularly pocket- and lead-related issues. Therefore, patients who receive leadless pacemakers may feel more independent, potentially requiring less perceived social support.^7^ Also, this study included a conventional “face-to-face” follow-up, which allows for a comprehensive interaction with health professionals, and did not include follow-up via telemonitoring. This means that some PPM recipients may have missed appointments or had limited access to health care, especially those living in remote areas. Other potential confounders may be frailty, which affects functional status, as well as socioeconomic factors, lifestyle modifications, and patients’ perceptions.

### Study limitations

The cross-sectional design of this study precludes any inference of causality. Participants were recruited via convenience sampling at a single center, which may introduce selection bias and limit generalizability. No a priori power calculation was performed, and the sample size was determined by patient availability, potentially reducing the ability to detect smaller but clinically meaningful differences. Nevertheless, the absence of a power calculation may limit the ability to detect smaller, yet potentially clinically significant, differences, and this limitation is acknowledged. Patient-reported measures may be subject to recall or social desirability bias, and unmeasured confounders, including socioeconomic factors, frailty, living situation, depression/anxiety, and caregiver presence, could have influenced the observed outcomes. Data were collected at a single post-implantation time point, limiting insight into changes in perceived social support over time. Pre-implantation assessments were not performed, which could have provided additional context for understanding device acceptance and social support dynamics. Interviews were used for data collection, introducing potential interviewer and social desirability biases. The study population was limited to patients attending a single tertiary hospital cardiology clinic, restricting comparability with other clinical settings, healthy controls, or recipients of alternative cardiac devices such as leadless pacemakers. Information on device manufacturer, lead type, and remote monitoring was not collected, although these factors may influence perceptions of support and independence. Finally, key variables, including device acceptance and perceived level of information about health, were assessed using self-reported measures rather than validated instruments. While this approach captured subjective experiences, it may limit measurement validity and introduce potential response bias. Future research would benefit from larger, multicenter studies using standardized, validated instruments and including pre- and post-implantation assessments to better evaluate social support dynamics.

### Future perspectives

Social support as perceived by PPM recipients is an innovative strategy to facilitate device management. Health care professionals ought to plan interventions to address types of social support and activate available resources. In cases of limited support, it is essential to encourage patients’ participation in support or peer groups, which offer opportunities to make friends, broaden their network, and keep their identity. Re-evaluation of support through home care and follow-up visits may enlighten health care providers about the aspects overlooked. As pacemaker technology evolves, recipients are expected to experience improved device functionality and daily living support. Therefore, it is crucial to implement interventions that strengthen support networks, ensuring meaningful interactions.

## Conclusions

The results demonstrated that patients experience greater support from significant others and family and less support from friends.

Regarding factors associated with perceived social support, the current findings illustrated reduced levels of support from significant others among patients aged >85 years and single or divorced patients. Single or divorced patients, those with a pacemaker implanted for 2–4 years, and those who felt “poorly” informed about their health reported less support from family. Patients aged <75 years, single or divorced patients, and those who reported acceptance of their device reported greater support from friends, while patients with comorbid conditions reported less support from friends.

The current findings demonstrate the need to enhance knowledge regarding perceived social support in the field of pacing and to build a strong support system between health care providers, patients, family, relatives, and significant others. These findings highlight the importance of considering social support when managing elderly patients with pacemakers, although further multicenter longitudinal studies are needed.
